# Anesthetic Challenges in Ameloblastoma of the Mandible: A Case Report

**DOI:** 10.7759/cureus.66741

**Published:** 2024-08-12

**Authors:** Sonal Khatavkar, Nayana Raveendran

**Affiliations:** 1 Anaesthesiology, Dr. D. Y. Patil Medical College, Hospital and Research Centre, Dr. D. Y. Patil Vidyapeeth, Pune, IND

**Keywords:** oral surgery, difficult airway, tumours, paediatric surgery, paediatric anaesthesia

## Abstract

Ameloblastoma is a rare, locally aggressive benign tumor primarily affecting the mandible, with an incidence of 0.92 cases per million person-years and a male predominance.

A two-year-old male presented with a right mandibular mass. CT imaging and histopathology confirmed ameloblastoma. He underwent wide local excision, mandibulectomy, and pectoralis major myocutaneous flap reconstruction under general anesthesia. Preoperative assessment revealed potential airway challenges; intubation was achieved with the backward, upward, rightward pressure (BURP) maneuver, and tracheostomy was performed to secure postoperative airway patency. The surgery was uneventful, and the patient was successfully weaned off ventilatory support by postoperative day four. This case underscores the importance of careful planning and expertise in pediatric ameloblastoma management, highlighting the effectiveness of direct laryngoscopy with BURP maneuver and prophylactic tracheostomy.

## Introduction

Ameloblastoma is a slow-growing but locally aggressive benign tumor that comprises 10% of the odontogenic tumors [1,2]. It arises from either odontogenic cyst epithelium or residual epithelium in remnant enamel over the crown of unerupted tooth. Incidence is 0.92 cases per million person-years [3] and is prevalent in all age groups though very rarely seen in the pediatric age group [4-6] with predominance in males as compared to females [7]. It is usually diagnosed radiologically. Goals of the treatment are complete tumour resection with functional and aesthetic restoration.

## Case presentation

**Figure 1:**
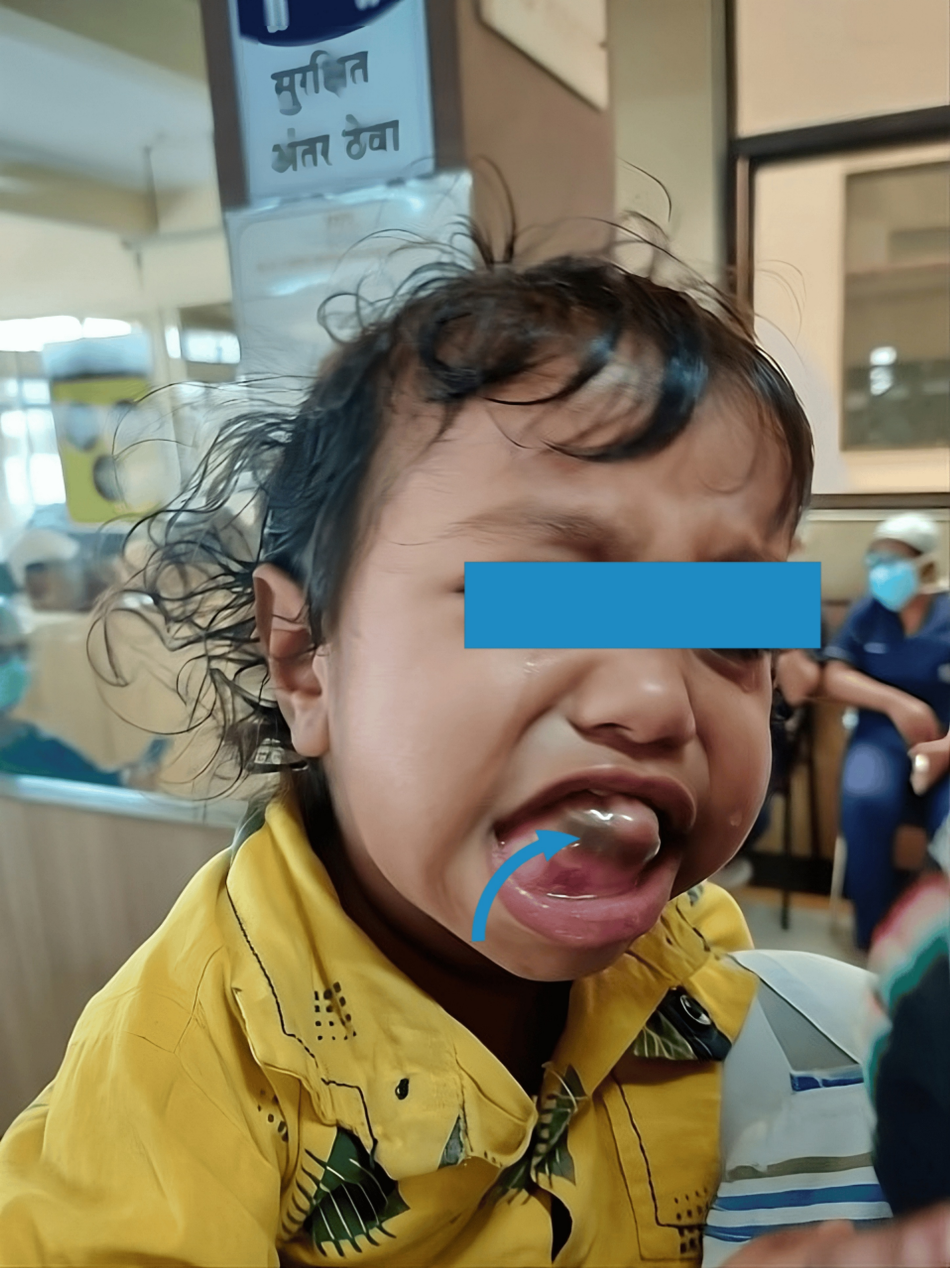
Solitary diffuse swelling over right mandible (arrow)

A two-year-old male child of weight 12kg was brought to the Department of Oral and Maxillofacial Surgery of our institution with the complaint of a mass protruding from lower jaw since birth which has gradually increased to the present size. No history of discharge from the swelling or local rise in temperature. On examination, there was a solitary diffuse swelling over right mandible (Figure 1) measuring about 40x40 mm. It was non-tender and hard on palpation. There was missing tooth over the mass.

Investigations

Computed Tomography (CT) scan of the mandible revealed a well-defined, expansile lesion of size approximately 40mm* 40mm*38mm in the body and part of ramus of right hemimandible. The cystic leision showed with multiseptated cystic spaces, ground glass ossified matrix within. The lesion extends anteriorly to involve symphysis menti and medial part of the body of the left hemimandible and posteriorly up to the left second molar tooth area. Bilateral temporo-mandibular joints, nasal bones, sinuses and orbits appeared normal.

Treatment

An incisional biopsy was performed two weeks prior under general anaesthesia (nasal intubation) which was uneventful. The specimen was subjected to histopathological examination which revealed it be ameloblastoma. As the lesion was extensive, wide local excision with mandibulectomy with pectoralis major myocutaneous (PMMC) flap reconstruction under general anaesthesia was planned.

A thorough preoperative evaluation was done, which consisted of detailed history about the onset and progression of the mass. Birth history, medical history and physical examination were unremarkable except for the orofacial findings. Mouth opening was adequate but Mallampati score couldn’t be assessed with a mass protruding from the lower jaw. Bilateral temporomandibular joint movement and cervical spine was normal. We were concerned about the difficult airway pertaining to inadequate seal with the mask during bag and mask ventilation and difficulty in laryngoscopy. So we prepared the difficult airway cart. In the operating room, all standard monitors, ECG, non-invasive blood pressure (NIBP), and saturation probe were attached and end tidal carbon dioxide monitor was kept ready. Nil by mouth for six hours was confirmed and consent was checked. Premedication with injection glycopyrolate 4 mcg/kg, injection midazolam 20 mcg/kg, injection ketamine 1 mg/kg intravenously were given. After preoxygenating with 100 percent oxygen for five minutes, induction was done with injection propofol 3 mg/kg and sevoflurane at 1.5% with spontaneous ventilation. On direct laryngoscopy with macIntosh blade larynx was not fully visualised, Cormack Lehane grade 4 was noted. So with a trained assistants’ aid, good backward upward rightward pressure (BURP) maneuver was performed to improve the glottic view, which converted the Cormack Lehane grade to 3 following which a bougie was passed and the endotracheal tube was railroaded over it. In case, if BURP was of no help the alternate plans to preserve the airway were 1) paediatric fibreoptic under spontaneous inhalational method, and 2) tracheostomy which is the gold standard method. Position of the tube was confirmed with auscultation and capnography. Throughout the induction and intubation procedure, oxygen saturation remained normal. Anticipating distortion of facial contour, airway edema and possibility of tongue fall, predisposing to difficulty in maintaining the patency of the airway in the postoperative period, tracheostomy was done before proceeding with the surgery. Intraoperatively patient was maintained on pressure control mode of ventilation with a 1:1 fresh gas mixture of oxygen and air, sevoflurane and intermittent doses of injection atracurium. Intraoperative hemodynamic and ventilatory parameters remained stable, adequate hydration with fluids was maintained, blood loss was replaced with blood products. Urine output was adequate.

Outcome and follow-up

The surgery was uneventful. Patient was shifted to Pediatric Intensive Care Unit on ventilatory support through tracheostomy. Weaning off was started from postoperative day one. He was maintaining saturation on room air through the tracheal stoma by day four.

## Discussion

It is prudent to anticipate and accept the airway challenges posed by en bloc removal of the tumour and the newly constructed mandible since it is a rarely performed surgery in the pediatric age group. Tumour growth distorts facial contour causing inadequate mask seal precluding bag and mask ventilation during induction, difficulty in laryngoscopy causing inability to visualise vocal cords [8]. We chose to proceed with direct laryngoscopy over awake fibre optic bronchoscopy. Shinha et al. have reported that oral fiberoptic bronchoscopy failed to visualize the glottis and epiglottis in a child with a large parapharyngeal mass while the glottis was visualised in the first attempt using C-Mac video laryngoscope [9]. The BURP maneuver facilitates improving the view of the glottis during difficult laryngoscopy as described by Takahata et al. [10].

It is challenging to maintain patency of the airway in the immediate postoperative period, because the newly constructed mandible predisposes to tongue fall and risk of significant airway oedema. Hence we chose tracheostomy to secure the airway as it’s a safer option in pediatric population, anticipating airway oedema, with possibility of tongue fall and risk of aspiration in the postoperative period.

## Conclusions

Our case report illustrates management of difficult ventilation and intubation and maintaining airway patency in the postoperative period in a patient with a massive ameloblastoma. The anaesthesiologists should be prepared with the difficult airway cart and be aware of the possibility of failed intubation with direct laryngoscopy and plan for alternative approaches to secure the airway whenever difficult intubation is anticipated.
